# Chinese Herbal Medicine Formula Guizhi Li-Zhong Tang as an Alternative to Antibiotic Feed Additives for Preventing Pneumonia in Piglets through Antioxidant, Anti-Inflammatory, and Antiapoptotic Protection

**DOI:** 10.1155/2021/4978783

**Published:** 2021-09-07

**Authors:** Che-Hsuan Wang, Wan-Jhen Wu, Li-Yu Su, Chen-Wen Lu, Pei-Hwa Wang, Ming-Chung Lee, Wu-Chang Chuang, Sheue-Er Wang, Chung-Hsin Wu

**Affiliations:** ^1^School of Life Science, National Taiwan Normal University, Taipei, Taiwan; ^2^Department of Animal Science and Technology, National Taiwan University, Taipei, Taiwan; ^3^Brion Research Institute of Taiwan, New Taipei, Taiwan; ^4^Sun Ten Pharmaceutical Co. Ltd, New Taipei, Taiwan; ^5^Pathological Department, Saint Paul's Hospital, Taoyuan, Taiwan

## Abstract

Weanling piglets often develop respiratory diseases such as pneumonia because they encounter substantial environmental stress. This study investigated an alternative herbal feed additive, Guizhi Li-Zhong Tang (GLZ), for preventing pneumonia in weanling piglets. An in vitro experiment demonstrated that GLZ has high antioxidant capacity and low cytotoxicity toward Kupffer cells. In addition, GLZ treatment can alleviate lipopolysaccharide (LPS)-induced damage in Kupffer cells. A total of 94 4-week-old piglets were randomly divided into three groups, which received sham treatment, 0.2% Tilmicosin antibiotic (TAB) treatment, or 0.2% GLZ treatment. Piglets receiving the GLZ treatment had a higher survival rate and higher immunoglobulin G levels but lower allergy-related eosinophil levels and cough incidence than did piglets receiving the sham or 0.2% TAB treatments. Through immunohistochemistry and Western blot analysis, we discovered that piglets receiving the 0.2% GLZ treatment had significantly higher expression of antioxidant-related SOD2 and lower expression of oxidative-stress-related 3-NT (*p* < 0.01), inflammation-related TNF-*α* (*p* < 0.01) and NF-*κ*B (*p* < 0.05), and apoptosis-related caspase-3 (*p* < 0.01) in lung tissue than did piglets receiving the sham or 0.2% TAB treatment. Therefore, GLZ treatment is promising as an alternative to antibiotic medicine for weanling piglets because of its protective antioxidative, anti-inflammatory, and antiapoptotic effects in lung tissue.

## 1. Introduction

Respiratory diseases in pigs are a major health problem in animal husbandry. Porcine respiratory diseases are usually the result of both primary and opportunistic infectious agents [1]. Porcine respiratory disease syndrome is an economically significant respiratory disease characterized by reduced feed efficiency, slow growth, cough, fever, and difficulty breathing [2]. Respiratory disease in pigs often results in pneumonia complicated by opportunistic bacterial infections. Oxidative stress is key for the development and progression of pneumonia because of the release of reactive oxygen species (ROSs) from alveolar macrophages and neutrophils in inflammatory lung tissue [3, 4]. ROSs cause the oxidation of proteins, DNA, and lipids, possibly leading to lung injury. Increased oxidative stress accompanied by reduced endogenous antioxidant defenses may cause inflammatory pulmonary diseases such as asthma.

Weanling piglets often suffer from respiratory diseases such as pneumonia because they encounter substantial environmental stress, such as dietary changes or infections that lead to digestive issues or slow growth [5–8]. Therefore, antibiotics are often added to feed for weanling piglets to promote growth and prevent respiratory diseases [9, 10]. Because of concerns regarding antibiotic residues and bacterial antibiotic resistance, the development of alternative feed additives for preventing respiratory diseases in piglets is critical [11, 12]. Herbal extracts can be used as alternative medicines because of their antimicrobial activity and antiviral properties [13–16]. Herbal extract preparations are widely used to treat childhood pneumonia in China [17].

Recent studies have indicated that weanling piglets often experience pneumonia with a high expression of angiotensin-converting enzyme 2 (ACE-2). ACE-2, a key receptor in SARS-CoV-2, was associated with the outbreak of COVID-19 [18]. In patients with COVID-19, high ACE-2 expression was identified in lung alveolar cells, and some patients had severe pneumonia [19]. We previously discovered that Guizhi Li-Zhong Tang (GLZ) could be used as an alternative feed additive to significantly reduce ACE-2 expression in the lung tissue of piglets as compared with sham or antibiotic treatments [20]. Although GLZ can be used to treat pneumonia in weanling piglets by reducing ACE-2 expression in lung tissue, the related protective mechanisms have not been studied systematically. This study investigated the mechanisms of GLZ as a feed additive for protection against pneumonia in the lung tissue of weanling piglets.

## 2. Materials and Methods

### 2.1. GLZ Herbal Formula Preparation

In this study, GLZ was selected as an alternative feed additive that has obtained a Taiwan patent (NTNU-P10843). GLZ is mainly composed of Guizhi and Li-Zhong Tang in specific proportions, and the dosage is 1-2 kg of GLZ per 1,000 kg of feed. All Chinese medicine materials are purchased from Sun-Ten Pharmaceutical Company (New Taipei City, Taiwan). The medicinal ingredients in weight percentage are as follows: Guizhi 5–40%, Ginseng 10–25%, licorice 10–25%, *Atractylodes macrocephala* 10–35%, and ginger 10–25%. As our previous study [20], we analyzed bioactive marker compounds of GLZ through three-dimensional chromatography.

### 2.2. Assays of Kupffer Cells after 0.2% GLZ Treatment

The antioxidant activity of GLZ was assessed through a 1,1-diphenyl-2-picrylhydrazyl (DPPH, D9132, Sigma-Aldrich, St. Louis, MO, USA) assay. The DPPH assay method was similar to that used in our previous research [21]; the DPPH free radical scavenging of the 0.2% GLZ treatment was analyzed using a mixture of DPPH in methanol. The cytotoxicity to Kupffer cells of the 0.2% GLZ treatment was assessed by using a3-(4,5-dimethylthiazol-2-yl)-2,5-diphenyltetrazolium bromide (MTT, M5655, Sigma-Aldrich Co., St. Louis, MO, USA) assay. Kupffer cells (SCC119) were obtained from Sigma-Aldrich Co. (St. Louis, MO, USA). After 24 hours of 0.2% GLZ treatment, the viability of Kupffer cells was analyzed and quantified using MTT assay with or without lipopolysaccharide (LPS) (L-2654, Sigma-Aldrich) treatment.

### 2.3. Animal Preparation

A total of 94 male weanling piglets were randomly divided into three groups, which received sham, 0.2% Tilmicosin antibiotic (TAB), or 0.2% GLZ treatment, and reared from 4 to 10 weeks of age at Dafeng Ranch (Yunlin County, Taiwan) under pathogen-free conditions at a constant temperature of 25°C with a 24 h light-dark cycle. At 10 weeks of age, a total of 12 weanling piglets (three from each group) were selected randomly and sacrificed to take lung tissue samples. Our piglet experiments followed the 3R (replacement, reduction, and refinement) principles and the Guidelines for the Care and Use of Laboratory Animals (permit number: NTNU/Animal Use/No. 109007).

### 2.4. Breeding Rate and Blood Immunoglobulin

We recorded changes in the breeding rate once every 2 days from 4 to 10 weeks of age in the sham, 0.2% TAB, and 0.2% GLZ treatment groups. At 10 weeks of age, blood samples were collected from the piglets in each group. By using Sandwich immunoglobulin G (IgG) enzyme-linked immunosorbent assay kits (E4472-100 and E4476-100; BioVision, Milpitas, CA, USA), blood IgG levels were analyzed on a Multiskan GO Microplate Spectrophotometer reader (Thermo Scientific, Waltham, MA, USA) according to the manufacturer's instructions.

### 2.5. Cough Incidence and Hematology

Through continuous video and audio analysis obtained from cameras that were set up above the pig house, we calculated and recorded the incidence of cough from 4 to 10 weeks of age for all treatment groups. At 10 weeks of age, hematology analysis of the piglets with each treatment was performed using a ProCyte Dx Hematology Analyzer (IDEXX Laboratories, Westbrook, ME, USA), which is an automated hematology analyzer that evaluates 24 parameters in an animal blood sample in approximately 2 minutes.

### 2.6. Immunohistochemical Staining

At 10 weeks of age, lung tissue was collected from the piglets in each group. The lung tissue was fixed in 4% formaldehyde and embedded in paraffin. The lung tissue samples were cut into 5 *μ*m sections by using a microtome, and these sections were then mounted on a glass slide for staining. For hematoxylin and eosin (H&E) staining, a kit (Sigma-Aldrich Corporation, St. Louis, MO, USA) was used for lung tissue morphology. Immunohistochemical (IHC) staining was used with antibodies for SOD2, 3-NT, TNF-*α*, NF-*κ*B, and caspase-3 (Cell Signaling Technology, Danvers, MA, USA) to evaluate their expression in lung tissue. Additionally, 3,3′-diaminobenzidine chromogen (Novolink Polymer Detection System l) was used, followed by counterstaining with hematoxylin (Novolink Polymer Detection System l).

### 2.7. Western Blotting

The lung tissue from the piglets was extracted using a buffer similar to that used in our previous research. Extracted lung proteins were separated using 10% sodium dodecyl sulfate-polyacrylamide gel electrophoresis (Bionovas Inc., Toronto, Ontario, Canada) and blotted onto a nitrocellulose membrane. The membrane was treated with SOD2, 3-NT, TNF-*α*, NF-*κ*B, caspase-3, and *β*-actin antibodies (Thermo Fisher Scientific Inc.). Secondary antibodies and chemiluminescent substrates (GE Healthcare Life Sciences, USA, Barrington, IL, USA) were also used to induce chemiluminescence that was detected by an LAS-4000 (GE Inc. Healthcare Life Sciences). The optical density of the Western blot was evaluated in ImageJ (version 1.48 t, NIH, Washington, DC, USA).

### 2.8. Statistical Analysis

Blood IgG levels, eosinophil percentage, cough incidence, and the relative expression of SOD2, 3-NT, TNF-*α*, NF-*κ*B, and caspase-3 are expressed as mean ± standard error. Statistical analysis was conducted using one-way analysis of variance followed by the Student–Newman–Keuls multiple-comparison posttest. *Ap* value of <0.05 was considered significant.

## 3. Results

### 3.1. Antioxidative Capacity and Kupffer Cell Viability

**Figure 1:**
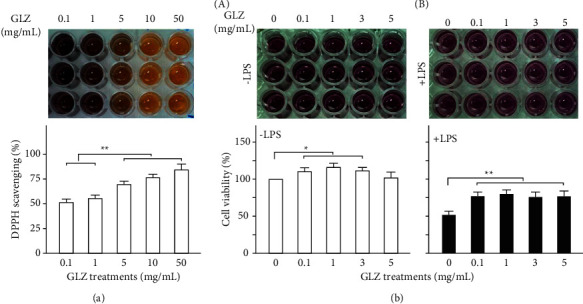
Antioxidant capacity and cytotoxicity of GLZ treatment. (a) DPPH free radical scavenging activity increased significantly with GLZ concentrations. (*n* = 3 for each group). (b) MTT assay under various concentrations of GLZ treatments without (A) -LPS) and with (B) +LPS) LPS treatment. Irrespective of LPS treatment, Kupffer cell viability for GLZ treatments (5–20 mg/mL) was significantly greater than for sham treatment (*n* = 3 for each group). Data are shown as mean ± standard error of the mean (^*∗∗*^*p* < 0.01, one-way analysis of variance followed by Student–Newman–Keuls multiple-comparison posttest).

The antioxidant capacity and cytotoxicity of GLZ were determined through DPPH and MTT assays, respectively.  Figure 1(a) displays the DPPH free radical scavenging activity in each group that was significantly greater at higher GLZ concentrations (*p* < 0.01). The results reveal that GLZ treatment has high antioxidant capacity and thus can scavenge for damaging free radicals.  Figure 1(b) A reveals that the Kupffer cell viability that GLZ treatment has low cytotoxicity to Kupffer cells (*p* < 0.05). Thus, 0.2% GLZ treatment may be suitable as an antibiotic medicine for weanling piglets because its antioxidant activity and low cytotoxicity. As shown in Figure 1(b) B, GLZ treatment can alleviate LPS-induced damage to Kupffer cells (*p* < 0.01).

### 3.2. Survival Rate, Blood IgG Levels, and Pneumonia

**Figure 2:**
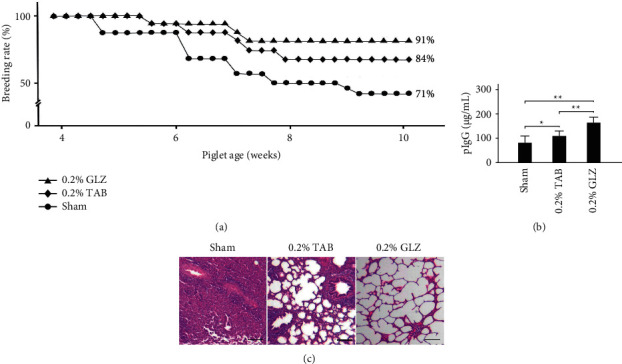
GLZ treatment increases survival rate and blood immunoglobulin IgG levels and prevents pneumonia in weanling piglets. (a) Changes in survival rate among piglets from 4 to 10 weeks of age receiving sham, 0.2% TAB, and 0.2% GLZ treatments. (b) Blood IgG levels in piglets at 10 weeks of age for each treatment. Values are mean ± standard error of the mean (*n* = 3 for each group; ^*∗∗*^*p* < 0.01, ^*∗*^*p* < 0.05, one-way analysis of variance followed by Student–Newman–Keuls multiple-comparison posttest). (c) Representative H&E stains of lung tissue of piglets at 10 weeks of age for each treatment. Pneumonia was observed in lung tissue in sham and 0.2% TAB groups but not in 0.2% GLZ group. Scale bars = 150 *μ*m.

Figure 2(a) displays the changes in survival rates among the weanling piglets receiving sham, 0.2% TAB, and 0.2% GLZ treatments from 4 to 10 weeks of age. The survival rate for the piglets receiving the sham treatment was lower than those receiving the 0.2% TAB treatment and 0.2% GLZ treatment.  Figure 2(b) presents the blood IgG levels in the blood of the piglets at 10 weeks of age for each treatment. The piglets receiving 0.2% GLZ treatment had significantly (*p* < 0.01) higher blood IgG levels than did the piglets receiving the sham or 0.2% TAB treatment and thus probably have superior immune function.  Figure 2(c) displays the H&E lung tissue stains for the weanling piglets at 10 weeks of age after receiving the sham, 0.2% TAB, and 0.2% GLZ treatments. Pneumonia was observed in the lung tissue of piglets receiving the sham and 0.2% TAB treatments but not observed in the lung tissue of the piglets receiving the 0.2% GLZ treatment. These results are consistent with the higher survival rate and IgG levels observed in the piglets receiving 0.2% GLZ. Therefore, 0.2% GLZ treatment may be a useful antibiotic medicine for weanling piglets because of its ability to enhance immune function.

### 3.3. Allergy-Related Eosinophil Levels and Cough Incidence

**Figure 3:**
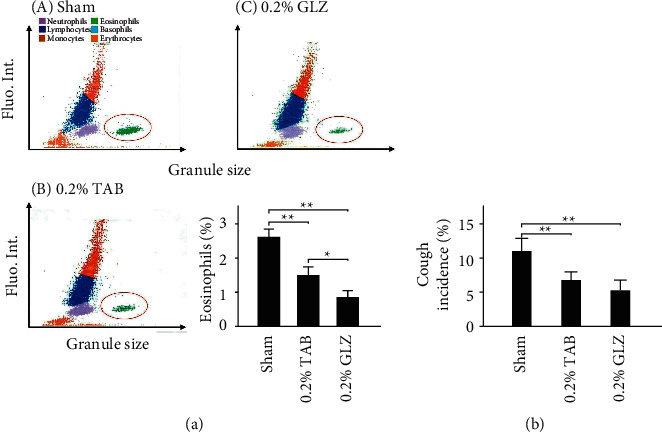
Allergy-related eosinophil levels and cough incidence. (a) Hematology analysis of piglets at 10 weeks of age that received (A) sham, (B) 0.2% TAB, and (c) 0.2% GLZ treatment. Allergy-related eosinophils are marked inside the red circle. Eosinophils of piglets from 4 to 10 weeks of age for each treatment (*n* = 3 for each group). (b) Cough incidence among piglets from 4 to 10 weeks of age receiving sham (*n* = 31), 0.2% TAB (*n* = 31), and 0.2% GLZ treatments (*n* = 32). Values are mean ± standard error of the mean (^*∗∗*^*p* < 0.01, ^*∗*^*p* < 0.05, one-way analysis of variance followed by Student–Newman–Keuls multiple-comparison posttest).

We examined allergy-related the eosinophil levels in the blood of 10-week-old piglets after each treatment. As Figure 3(a) presents, 0.2% GLZ treatment significantly reduced the level of allergy-related eosinophils in piglet blood (*p* < 0.01). We further examined the cough incidence among the piglets from 4 to 10 weeks of age.  Figure 3(b) reveals that the cough incidence results are consistent with those for the allergy-related eosinophil levels. Therefore, 0.2% GLZ treatment may be effective as an alternative medicine for attenuating respiratory allergies in weanling piglets.

### 3.4. Antioxidant-Related SOD2 and Oxidative-Stress-Related 3-NT Expression in Lung Tissue

**Figure 4:**
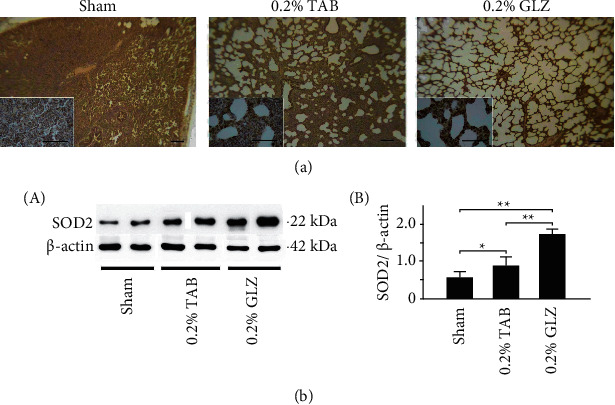
Herbal formula GLZ treatment can increase antioxidant-related SOD2 expression in the lung tissue of weanling piglets. (a) Representative IHC stains of SOD2 expression from piglets at 10 weeks of age after each treatment. SOD2 expression is indicated by dark brown. Scale bars are 30 *μ*m for large images and 150 *μ*m for small images. (b) Western blot for SOD2 expression in lung tissue of piglets at 10 weeks of age after each treatment (*n* = 3 for each group). Values are mean ± standard error of the mean (^*∗∗*^*p* < 0.01, ^*∗*^*p* < 0.05, one-way analysis of variance followed by Student–Newman–Keuls multiple-comparison posttest).

**Figure 5:**
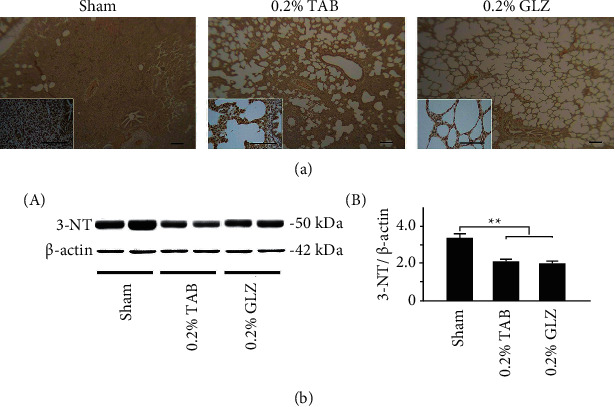
GLZ treatment reduced expression of oxidative-stress-related 3-NT in the lung tissue of weanling piglets. (a) Representative IHC stains of 3-NT expression for piglets at 10 weeks of age after sham, 0.2% TAB, and 0.2% GLZ treatments. 3-NT expression is indicated by dark brown. Scale bars are 30 *μ*m for large images and 150 *μ*m for small images. (b) Western blot for 3-NT expression in the lung tissue of piglets at 10 weeks of age after each treatment (*n* = 3 for each group). Values are mean ± standard error of the mean (^*∗∗*^*p* < 0.01, one-way analysis of variance followed by Student–Newman–Keuls multiple-comparison posttest).

To determine the antioxidant effects of the GLZ treatment, the expression of antioxidant-related SOD2 and oxidative-stress-related 3-NT was examined in 10-week-old piglet lung tissue. The IHC staining displayed in Figure 4(a) demonstrates that SOD2 expression in the lung tissue was high for the piglets receiving the GLZ treatment but low for those receiving the sham treatment. The results of the Western blot analysis are presented in Figure 4(b) and demonstrate that the antioxidant-related SOD2 expression in the piglets receiving the GLZ treatment was significantly higher than that in piglets receiving the sham or 0.2% TAB treatment (*p* < 0.01).  Figure 5(a) demonstrates that 3-NT expression in lung tissue was low for the piglets receiving the 0.2% GLZ treatment and higher for the piglets receiving the sham treatment.  Figure 5(b) shows that the oxidative-stress-related 3-NT expression of both the piglets receiving the 0.2% GLZ and TAB treatments was significantly lower than that of the piglets receiving the sham treatment (*p* < 0.01). Figures 4 and 5 reveal that 0.2% GLZ treatment alleviated oxidative stress in piglet lung tissue.

### 3.5. Inflammation-Related TNF-*α* and NF-*κ*B Expression in Lung Tissue

**Figure 6:**
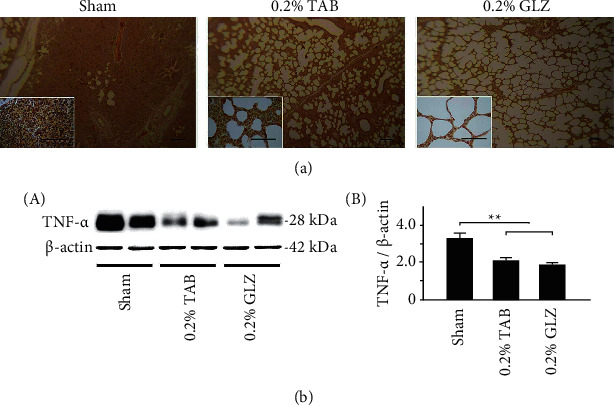
GLZ treatment reduced expression of inflammation-related TNF-*α* in the lung tissue of weanling piglets. (a) Representative IHC stains of TNF-*α* expression of piglets at 10 weeks of age after sham, 0.2% TAB, and 0.2% GLZ treatments. TNF-*α* expression is indicated by dark brown. Scale bars are 30 *μ*m for large images and 150 *μ*m for small images. (b) Western blot for TNF-*α* expression in the lung tissue of piglets at 10 weeks after each treatment (*n* = 3 for each group). Values are mean ± standard error of the mean (^*∗∗*^*p* < 0.01, one-way analysis of variance followed by Student–Newman–Keuls multiple-comparison posttest).

**Figure 7:**
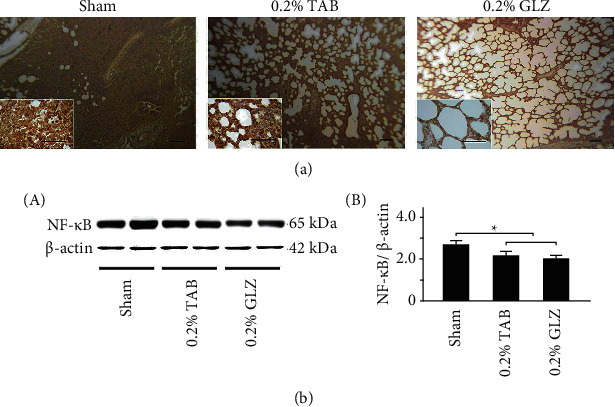
GLZ treatment reduced expression of inflammation-related NF-*κ*B in the lung tissue of weanling piglets. (a) Representative IHC stains for NF-*κ*B expression among piglets at 10 weeks of age after sham, 0.2% TAB, and 0.2% GLZ treatments. NF-*κ*B expression is indicated by dark brown. Scale bars are 30 *μ*m for large images and 150 *μ*m for small images. (b) Western blot for NF-*κ*B expression in the lung tissue of piglets at 10 weeks of age after each treatment (*n* = 3 for each group). Values are mean ± standard error of the mean (^*∗*^*p* < 0.05, one-way analysis of variance followed by Student–Newman–Keuls multiple-comparison posttest).

To determine the anti-inflammatory effects of GLZ treatment, the expression of the inflammation-related TNF-*α* and NF-*κ*B in the lung tissue of the 10-week-old piglets was examined for each treatment group.  Figure 6(a) reveals that the TNF-*α* expression in lung tissue was low for the piglets receiving the 0.2% GLZ treatment but high for those receiving the sham treatment. The results of Western blot analysis presented in Figure 6(b) reveal that the inflammation-related TNF-*α* expression of the piglets receiving the 0.2% GLZ treatment was significantly lower than that of the piglets receiving the sham and 0.2% TAB treatments (*p* < 0.01).  Figure 7(a) reveals that the NF-*κ*B expression in lung tissue was low for the piglets receiving the 0.2% GLZ treatment but high for those receiving the sham treatment.  Figure 7(b) shows that the inflammation-related NF-*κ*B expression in the piglets receiving the GLZ and 0.2% TAB treatments was significantly lower than that in the piglets receiving the sham treatment (*p* < 0.01–0.05). Figures 6 and 7 reveal that 0.2% GLZ treatment alleviated inflammation in piglet lung tissue.

### 3.6. Apoptosis-Related Caspase-3 Expression in Lung Tissue

**Figure 8:**
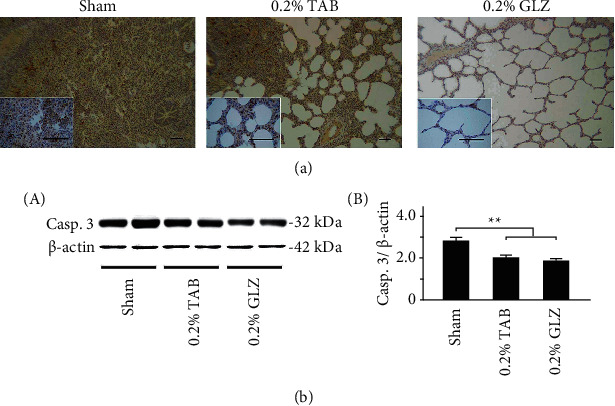
GLZ treatment reduced expression of apoptosis-related caspase-3 in the lung tissue of weanling piglets. (a) Representative IHC stains of caspase-3 expression among piglets at 10 weeks of age after sham, 0.2% TAB, and 0.2% GLZ treatments. Caspase-3 expression is indicated by dark brown. Scale bars are 30 *μ*m for large images and 150 *μ*m for small images. (b) Western blot for caspase-3 expression in the lung tissue of piglets at 10 weeks of age after each treatment (*n* = 3 for each group). Values are mean ± standard error of the mean (^*∗∗*^*p* < 0.01, one-way analysis of variance followed by Student–Newman–Keuls multiple-comparison posttest).

To determine antiapoptotic capacity of GLZ treatment, the expression of the apoptosis-related caspase-3 in the lung tissue was examined for the 10-week-old piglets in each treatment group. The IHC staining results are displayed in Figure 8(a); the caspase-3 expression in lung tissue was low for the piglets receiving the 0.2% GLZ treatment but high for the piglets receiving the sham treatment.  Figure 8(b) displays the results of the Western blot analysis that the apoptosis-related caspase-3 expression in the piglets receiving the GLZ and 0.2% TAB treatments was significantly lower than that in the piglets receiving the sham treatment (*p* < 0.01). The results in Figure 8 demonstrate that 0.2% GLZ treatment can alleviate apoptosis in piglet lung tissue.

## 4. Discussion

The herbal formula GLZ was investigated as an alternative medicine for weanling piglets. The formula can alleviate respiratory allergies and prevent pneumonia in weanling piglets by inhibiting ACE-2 expression in the respiratory system [20]. However, little is known regarding the underlying pharmacological mechanisms. Weanling piglets often experience respiratory diseases such as pneumonia because they encounter substantial environmental stress [5–8]. Therefore, antibiotics are often used in pig feed systems. However, antibiotic overuse can cause antibiotic resistance. The European Union banned the use of antibiotics in growth-promoter pig feed in 2006. Therefore, replacing antibiotic medicines with traditional Chinese medicine or natural products could have substantial benefits. The results of this in vitro experiment demonstrate that GLZ has high antioxidant capacity for scavenging free radicals (Figure 1(a)) and low Kupffer cell cytotoxicity (Figure 1(b)). Therefore, GLZ treatment may be useful as an alternative medicine for weanling piglets.

For weanling piglets, the herbal formula GLZ treatment can increase the survival rate (Figure 2(a)) and immunity related IgG levels (Figure 2(b)). Compared to piglets treated with GLZ treatment, those piglets with sham and TAB treatments showed varying degrees of pneumonia, higher allergy-related eosinophil levels (Figure 3(a)), and more frequent cough incidence (Figure 3(b)). Pneumonia in weanling piglets may be caused by respiratory infections. TAB treatment may kill harmful viruses, bacteria, parasites, and other microorganisms but may also kill probiotics in the gastrointestinal tract. By contrast, GLZ treatment has antioxidative properties and can improve the immune function of piglets, that is, their ability to resist harmful viruses, bacteria, parasites, and other microorganisms, without harming the beneficial probiotics in the gastrointestinal tract. This may explain why GLZ is preferable to TAB for preventing piglet pneumonia.

Through IHC and Western blot analysis of lung tissue, we observed that the piglets receiving the 0.2% GLZ treatment had significantly higher expression of antioxidant-related SOD2 (Figure 4) but lower expression of oxidant-related 3-NT (Figure 5), inflammation-related TNF-*α* (Figure 6) and NF-*κ*B (Figure 7), and apoptosis-related caspase-3 (Figure 8) than did the piglets receiving the TAB or sham treatment. Therefore, GLZ treatment may have protective antioxidative, anti-inflammatory, and antiapoptotic effects that alleviate pneumonia in the weanling piglet respiratory system.

A component of GLZ, Guizhi, is a traditional herbal preparation that has been used to treat head-related illnesses such as fever, headache, sweating, and wind-induced headaches [22]. Chromatographic investigation of Guizhi in our previous research [20] revealed that its bioactive substances are coumarin, cinnamic acid, cinnamaldehyde, and 2-methoxycinnamaldehyde. Coumarin has been used in various products and candidate drugs because coumarin compounds have high bioavailability and both antioxidant and anti-inflammatory properties [23]. Cinnamic acid and its derivatives (such as ferulic acid and cinnamic amide) have attracted substantial attention due to their antioxidative, anti-inflammatory, and neuroprotective properties [24]. Cinnamaldehyde, the major constituent of the bark of *Cinnamomum cassia*, has been reported to have antioxidative, anti-inflammatory, and antibacterial properties [25, 26]. 2-Methoxycinnamaldehyde also demonstrated antioxidative and anti-inflammatory properties [27]. Additionally, cinnamaldehyde and 2-methoxycinnamaldehyde were both identified as NF-*κ*B inhibitors [28]. NF-*κ*B is critical in the regulation of cell death, inflammation, and the immune responses that regulate the expression of chemokines such as TNF-*α* as well as of apoptotic proteins [29, 30].

The other component of GLZ, Li-Zhong Tang, was first mentioned in the traditional Chinese medicine scripture Treatise on Febrile Diseases 1800 years ago and is commonly used in traditional medicine to warm the “middle jiao” (i.e., the lower stomach), dispel cold, invigorate *qi* (life force), and strengthen the kidneys [31]. Chromatographic analysis of Li-Zhong Tang revealed that the bioactive marker compounds are liquiritin; glycyrrhizin; the ginsenosides Rg1, Re, and Rb1; atractylenolide III; 6-gingerol; and 6-shogaol [20]. Liquiritin, a major constituent of glycyrrhiza radix, may have exerted antioxidative, anti-inflammatory, and antiapoptotic effects through suppression of the NF-*κ*B and MAPK signaling pathways [32]. Glycyrrhizin, another bioactive component of glycyrrhiza radix, has reported antioxidative and anti-inflammatory properties in liver disease treatment [33]. Ginsenosides Rg1 and Re were reported to reduce oxidative stress and attenuate myocardial apoptosis in treatment of rats with diabetes [34]. Ginsenoside Rb1 has antioxidant and anti-inflammatory properties that can ameliorate acute lung injury by attenuating NF-*κ*B and MAPK activation [35]. Atractylenolide III, a bioactive component of Atractylodis Rhizoma, was reported to attenuate the transcriptional activity of NF-*κ*B and to treat inflammation-related neurodegenerative diseases [36]. 6-Gingerol is the most abundant active compound in fresh ginger, and 6-shogaol is the dehydrated form of 6-gingerol. Both 6-gingerol and 6-shogaol have antioxidant and anti-inflammatory properties [37], with those of 6-shogaol being superior. The DPPH free radical scavenging activity of 6-shogaol is also greater than that of 6-gingerol [38]. After reviewing all the bioactive marker substances in the herbal formula GLZ, we observed that almost all have antioxidant, anti-inflammatory, and antiapoptotic properties that may help to prevent or relieve pneumonia.

## 5. Conclusion

**Figure 9:**
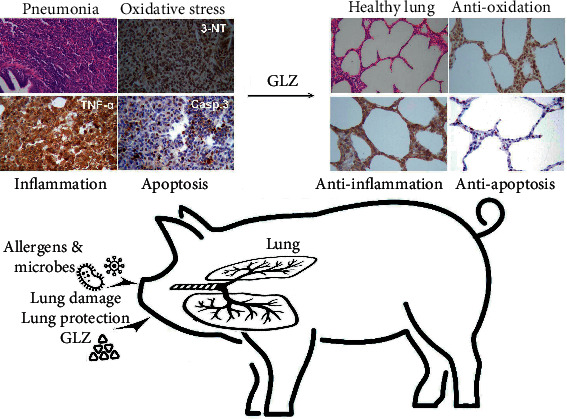
Diagram illustrating that GLZ treatment can protect the lungs from pneumonia in the respiratory system of weanling piglets by exerting anti-oxidant, anti-inflammatory, and anti-apoptotic effects in lung tissue.

In this study, as suggested in Figure 9, an alternative herbal medicine, GLZ, was investigated for preventing pneumonia in weanling piglets. Compared with antibiotic treatment, 0.2% GLZ treatment had greater antioxidant capacity and lower cytotoxicity, and weanling piglets receiving GLZ had a higher survival rate, greater immune function, milder allergies, and lower cough incidence than did those receiving antibiotics. GLZ treatment can prevent pneumonia in weanling piglets by exerting protective antioxidant, anti-inflammatory, and antiapoptotic effects. Therefore, the herbal medicine GLZ could be used as an alternative to antibiotic medicine for pneumonia in weanling piglets.

## Data Availability

The blood biochemical analysis, ROS analysis, immunohistochemistry, and Western blot analysis data used to support the findings of this study are included in the article and are available from the corresponding author upon request.
